# Odontalgia Cured by Ipecac

**Published:** 1892-11

**Authors:** 


### Odontalgia Cured by Ipecac.

Dr. Mossa, of Stuttgart, reports the case of a twenty-one year old and pale young man, of slender frame, who was much troubled with rheumatic pains. For eight days he had been afflicted with a very painful toothache. He had already had one tooth drawn, yet the pain had not ceased, but taken on another form. It proceeded from a molar tooth of the superior maxillary; it was piercing in character, and radiated into the right temple, ear and nose, and even into the other teeth. The pain would appear paroxysmally every few minutes with a jerk as though the teeth were being pulled out—worse by day than by night. The gums were, for some time, swollen and bleeding; this had been removed by nitric acid. His teeth were in bad condition in general; his tongue was coated and his appetite faulty.

The symptom, *paroxysmal and jerky pains* as though the tooth were being pulled out, led the writer to think of ipecac, of which the patient received several drops, three times a day, in a teaspoonful of water. The toothache and neuralgia soon ceased.—*Arch. fur Hom.*

